# *GLUL* gene knockdown and restricted glucose level show synergistic inhibitory effect on the luminal subtype breast cancer MCF7 cells’ proliferation and metastasis

**DOI:** 10.17179/excli2023-6287

**Published:** 2023-08-16

**Authors:** Arezu Karimpur Zahmatkesh, Mohammad Khalaj-Kondori, Mohammad Ali Hosseinpour Feizi, Behzad Baradaran

**Affiliations:** 1 Department of Animal Biology, Faculty of Natural Sciences, University of Tabriz, Tabriz, Iran; 2 Immunology Research Center, Tabriz University of Medical Sciences, Tabriz

**Keywords:** cancer metabolism, glutamine synthetase, glycolysis, luminal breast cancer, MCF7 cell line

## Abstract

The glutamine synthetase path is one of the most important metabolic pathways in luminal breast cancer cells, which plays a critical role in supplying glutamine as an intermediate in the biosynthesis of amino acids and nucleotides. On the other hand, glycolysis and its dominant substrate, glucose, are the most critical players in cancer metabolism. Accordingly, targeting these two critical paths might be more efficient in luminal-type breast cancer treatment. MCF7 cells were cultivated in media containing 4.5, 2, and 1 g/L glucose to study its effects on GLUL (Glutamate Ammonia Ligase) expression. Followingly, high and low glucose cell cultures were transfected with 220 pM of siGLUL and incubated for 48 h at 37 ºC. The cell cycle progression and apoptosis were monitored and assessed by flow cytometry. Expression of GLUL, known as glutamine synthetase, was evaluated in mRNA and protein levels by qRT-PCR and western blotting, respectively. To examine the migration and invasion capacity of studied cells exploited from wound healing assay and subsequent expression studies of glutathione-S-transferase Mu3 (GSTM3) and alfa-enolase (ENO1). Expression of GLUL significantly decreased in cells cultured at lower glucose levels compared to those at higher glucose levels. siRNA-mediated knockdown of GLUL expression in low glucose cultures significantly reduced growth, proliferation, migration, and invasion of the MCF7 cells and enhanced their apoptosis compared to the controls. Based on the results, GLUL suppression down-regulated GSTM3, a main detoxifying enzyme, and up-regulated Bax. According to the role of glycolysis as a ROS suppressor, decreased amounts of glucose could be associated with increased ROS; it can be considered an efficient involved mechanism in this study. Also, increased expression of Bax could be attributable to mTOR/AKT inhibition following GLUL repression. In conclusion, utilizing GLUL and glycolysis inhibitors might be a more effective strategy in luminal-type breast cancer therapy.

See also Figure 1(Fig. 1).

## Introduction

Breast cancer is the most common type of cancer and the second cause of cancer-related death in women (Bray et al., 2018[5]; Miller et al., 2020[46]; Redig and McAllister, 2013[52]; Smith et al., 2017[59]), which implies the need for improved therapeutic strategies (Li et al., 2020[39]). One of the most important hallmarks of cancer cells is metabolic reprogramming (Faubert et al., 2020[19]; Hanahan and Weinberg, 2011[25]; Pavlova and Thompson, 2016[50]; Revathidevi and Munirajan, 2019[54]). Determination of metabolic differences in cancerous and normal cells and their causal mechanisms will not only raise our knowledge of cancer cell biology but also present a significant basis for developing novel therapeutic approaches to devastate cancer cells (Altman et al., 2016[1]; Leone et al., 2019[37]; Luengo et al., 2017[45]; Vander Heiden and DeBerardinis, 2017[68]). Despite numerous studies, metabolic changes in cancer cells have not been determined thoroughly because of the complicated behavior of cancer cells and technical drawbacks (Baghban et al., 2020[3]). Nevertheless, metabolic reprogramming is known not only as an energy resource for the survival and proliferation of malignant cells but also plays some roles in the invasiveness and metastasis of cancer cells (Faubert et al., 2020[19]). Among different metabolic pathways in cancer cells, the metabolic networks of amino acids are very complicated and highly interlocked with other paths. Since malignant cells generally use an excessive amount of glutamine to ease rapid proliferation (Eagle et al., 1956[18]), the metabolic pathway of glutamine has attracted the attention of more cancer researchers. Glutamine is the second vital nutrient for cancer cells after glucose (Kuo et al., 2000[36]). It is considered a critical source of precursors such as carbon and nitrogen in the biosynthesis of nucleotides, lipids, glutathione, intermediates of the tricarboxylic acid cycle, and others (Deprez et al., 1997[15]; Hanahan and Weinberg, 2011[25]; Warburg, 1956[72]; Wieman et al., 2007[74]). Mammalian cells generally synthesize glutamine via glutamine synthetase, *de novo* (Yuneva et al., 2007[77]). On the other hand, most cancerous cells, including non-small Cell Lung Cancer (Davidson et al., 2016[13]; Sellers et al., 2015[57]; Yuneva et al., 2012[78]), prostate (Fendt et al., 2013[20]), and pancreas cancer (Son et al., 2013[60]), fibrosarcoma (Reid et al., 2016[53]), and T cell ALL (Herranz et al., 2015[27]) rely on extracellular glutamine. Therefore, glutamine often is considered a conditionally essential amino acid (Wei et al., 2021[73]). On the other hand, the glutamine requirement of breast cancer is determined based on its molecular subtypes. Considering recent studies, the basal subtype of breast cancer is dependent on extracellular glutamine, and at the same time, the luminal subtype synthesizes glutamine, *de novo*, by glutamine synthetase (GS) (Kung et al., 2011[35]), which is also known as Glutamate Ammonia Ligase (GLUL). In HER2-positive and basal-like subtypes of breast cancer, the glutamine metabolism pathway demonstrates maximum activity where the expression of the glutaminase and glutamate dehydrogenase is enhanced but is substantially down-regulated in the luminal subtype (Cao et al., 2014[7]; Kanaan et al., 2014[32]; Kim et al., 2013[34]) and often used cytosolic glutamine amidotransferases for glutamine catabolism (Nguyen and Durán, 2018[47]). Accordingly, glutamine depletion of the cancer cell environment or inhibition of glutamine synthetase can affect cancer cell survival. Although glucose is one of the essential sources of energy in cancer and normal cells, the normal cells depend mainly on oxidative phosphorylation (OXPHOS) (Kalyanaraman, 2017[31]) but in cancer cells, aerobic glycolysis is the dominant mechanism that is known as the Warburg effect. Based on recent research, inhibiting glycolysis via enzymes or glucose transporters such as hexokinase and glucose transporter 1 (GLUT1) (Gautier et al., 2013[22]; Liu et al., 2012[41]), with or without chemical drugs (Jae et al., 2009[30]), however, suppresses the growth of the cancer cells, it is inadequate for eradication of tumor mass because these cells can adjust their metabolism to continue surviving in this situation (Ghanbari Movahed et al., 2019[23]). Accordingly, inhibiting more than one metabolic pathway for cancer treatment seems logical. Here we suppressed *GLUL* expression by siRNA and evaluated the simultaneous effects of *GLUL* knockdown and restricted glucose levels in MCF7 cells as a model for the luminal A breast cancer subtype.

## Materials and Methods

### Cell culture

The human breast cancer MCF7 cell line, ATCC HTB-22, was purchased from the National Cell Bank of Pasteur Institute, Iran, Tehran. Studied cells cultured in DMEM with high and low glucose and a medium with moderate glucose containing 4.5, 1, and 2 gr/liter of glucose, respectively (Gibco, Thermo Fisher, USA), supplemented with 10 % heat inactivated FBS (Anacell, Tehran, Iran), and 1 % of penicillin/streptomycin (10.000 U/mL) (Bioideaco, Tehran, Iran) in a moistened condition of 5 % CO_2_ at 37 °C.

### GLUL-specific silencing by small interfering RNA (siRNA)

GLUL-siRNA of interest (siGLUL) was synthesized by Bioneer, South Korea, with a sequence; 5′-GAUUGGACCUUGUGAAGGA-3**′ **(Guo et al., 2021[24]). For *GLUL* knockdown, the cultured MCF7 cells were detached and prepared to 220 pM siRNA delivery via electroporation with voltage 220 and capacitances 250 μF (modified condition to Michelle Colins' protocol) (Biorad, Gene Pulser Xcell™, UK). Following siGLUL transfection, cells were seeded in six-well plates at 2.2×10^5^ cells/well. Transfected and control cells detached and prepared for different investigations after 48 h incubation at 37 °C incubator.

### Quantitative real-time PCR (q-RT PCR)

Both cultured control and siGLUL transfected cells were detached from plates after 48 h with 0.25% EDTA-trypsin (DENAzist-Asia, Mashhad, Iran), and the total RNA was isolated using column RNA extraction kit (DENAzist-Asia, Mashhad, Iran) according to the company's instructions. The cDNA was subsequently synthesized with 1 μg of extracted RNA and by a cDNA easy synthesis kit for further specific studies (Parstoos, Mashhad, Iran). The quantitative RT-PCR was administered with primers for target genes, including *GLUL*, *GSTMu3*, *ENO1 *and *Bax*, and *β-actin* using High ROX RealQ Plus 2x Master Mix Green (AMPLIQON; Denmark) by ABI step one plus (Applied Biosystems, USA). Primer sequences and the conditions of qRT-PCR are available in Table 1(Tab. 1) (References in Table 1: Huang et al., 2019[29]; Kung et al., 2011[35]; Wang et al., 2020[70]). Studied genes were normalized based on the housekeeping gene β-actin as the reference gene. The relative expression values of mRNA were determined using the 2−∆∆Ct method between the siGLUL transfected and control cells.

### Western blotting

The total protein of cultured control and treated cells were extracted based on SDS, and the concentration of isolated protein was determined using the Bradford method (He, 2011[26]). Subsequently, denatured proteins, as the result of boiling in 100 °C water, were loaded on SDS-PAGE (sodium dodecyl sulfate-polyacrylamide gel electrophoresis) gels with a protein ladder (SuperSignal® Molecular Weight Protein Ladder, Thermo Fisher Scientific, USA). Separated proteins were transferred to the PVDF (polyvinylidene fluoride) membranes to isolate the target protein in a western blotting device; then the membranes were incubated with primary antibodies β-actin (sc-47778,1: 300), GAPDH (sc-47724, 1:300), and Glu synthetase (E-4) (sc-74430, Santa Cruz Biotechnology, 1:300) for 16-18 hours at 4 °C. Followingly, the PVDF membranes were washed with TBS-T buffer and shaken in a solution; containing secondary antibodies (anti-rabbit, 1:1000) for 75 minutes. Finally, the labeled proteins were visualized and investigated by chemiluminescence assay (ECL advanced reagents kit, Amersham, USA). The collected raw data were analyzed using Student's t-test. P value < 0.05 was considered statistically significant.

### Impact of levels of glucose and siGLUL on MCF7 Cell Morphology 

Moreover, the effects of various quantities of glucose and siGLUL transfection were investigated on cell morphology using Olympus's inverted microscope (10X).

### Flow cytometry analysis of cell cycle 

To study the cell cycle phases profited the flow cytometry technique (MACSQuant, USA). Target cells were cultivated in different amounts of glucose and evaluated for the cell cycle, and subsequently, based on the results, low glucose culture cells were treated by siGLUL (220 pm) and studied by flow cytometry. In this context, after 48 hours, the cells were detached and washed with phosphate-buffered saline (PBS) two times, fixed in 90 % ethanol at -23 °C overnight. The ethanol-fixed cells were centrifuged, washed with PBS, and stained with a solution containing PI and RNaseA at a final concentration of 0.001 mg/mL and 16.6 mg/mL, respectively. The prepared cells were incubated at 37 °C for 30 min and examined by FACS (MACSQuant, USA). Obtained Flow Cytometry data was analyzed using the FlowJo 10.8.1 software.

### Apoptosis evaluation with AnnexinV-FITC and PI staining 

To investigate the effect of *GLUL* knockdown on apoptosis, we utilize the AnnexinV - FITC and PI staining methods. GLUL siRNA was transfected, and cells were seeded and cultured for 72 hours. Followingly, the cells were detached with trypsin, washed with PBS three times, centrifuged, and resuspended in 197 μL binding buffer. According to the manufacturer's instructions (Immunostep, Spain), a compound of Annexin-V-FITC (3 μL) and PI (2.7 μL) was added and mixed into the cell suspension. Labeled cells were incubated for 15 minutes at room temperature in a dark compartment and studied by flow cytometry (MACSQuant, USA). The data were analyzed and evaluated using FlowJo 10.8.1 software.

### Cell migration assessment

The rate of migration in studied cells was examined and evaluated by two methods, including; a) wound healing assays and b) gene expression studies of Glutathione S-Transferase Mu-3 (*GSTM3*) and Alfa-enolase 1 (*ENO1*) (Dalla et al., 2020[11]). 

### Wound healing assay

In this trial, siGLUL transfected also not transfected cells were seeded in 12 wells plates with 1.1×10^5^ cells and placed in a 37 °C and 5 % CO_2 _incubator for 24 hours (Wang et al., 2019[71]). Followingly, the cell's adhesion was evaluated and then created a horizontal scratch in the middle of the plates using a 100 μL sterile pipette tip. Subsequently, scratched wells were washed with 1X PBS away with shaken gently for 30 seconds. Eventually, scratches were studied using an inverted microscope (Olympus CKX41) on a 10X objective and a camera at 0, 24, and 48 hours. The captured images were examined and analyzed with Fiji-ImageJ software. For further investigation, the expression of two known luminal breast cancer metastatic genes, including *GSTM3* and *ENO1*, was assessed (Dalla et al., 2020[11]).

### Statistical analysis

The software Graph PadPrism 9 and SPSS 28 were applied to analyze all collected data by Student's t-test and one-way ANOVA. A P value less than 0.05 was regarded as significant, statistically. All experiments were performed at least three times in the same situations, and the results were finalized and expressed by mean ± SEM (standard error of the mean).

## Results

### Down-regulation of GS (glutamine synthetase) expression in mRNA and protein levels by siGLUL transfection

**Figure 2:**
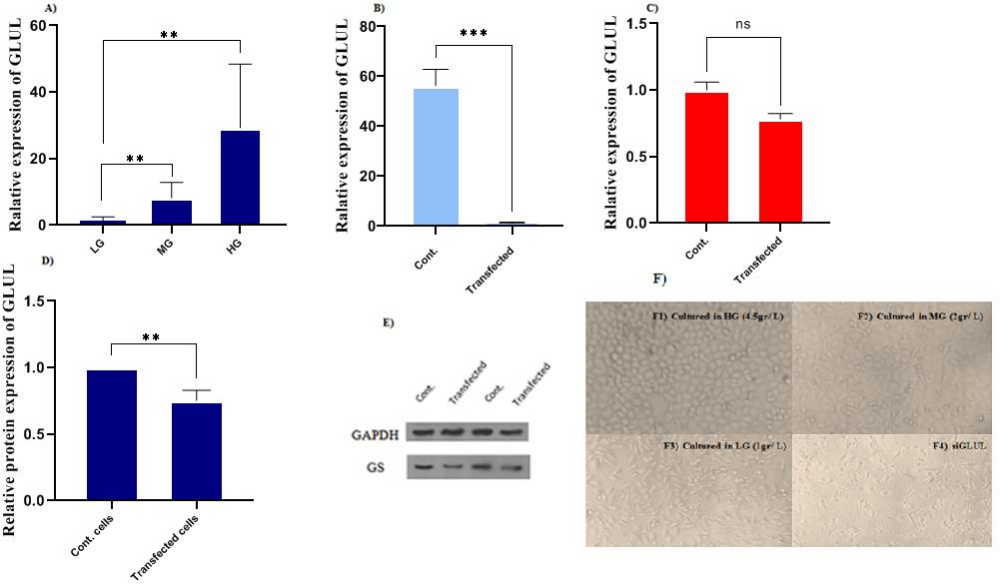
A) Expressions of *GLUL* (glutamine synthetase) mRNA in breast cancer MCF7 cells in a growth medium with different levels of glucose, low levels of glucose are associated with *GLUL* down-regulation compared to high and moderate levels of glucose ( P-value= 0.0071), B) Expressions of *GLUL *(glutamine synthetase) mRNA in control cells and transfected cells (with 220 nm of siRNA), which were cultured in low glucose DMEM, and C) high glucose DMEM, respectively. The results indicate a significant difference in expression between the control and transfected groups in cells cultured in low glucose DMEM (P=0.0001), D) Knockdown of *GLUL* and protein expression in MCF7 cells. Relative protein expressions of glutamine synthetase in siGLUL transfected and control MCF7 cells cultured in low glucose (1 gr/ liter) DMEM (p=0.0048). E) Western blot analysis of GLUL siRNAs transfected and control cells demonstrate efficient glutamine synthetase protein knockdown. F) Cell morphology studies in cells cultured in various mediums or treated (F1-F4). Cells cultured in high glucose DMEM (F1), Cells cultured in medium with moderate glucose (F2), Cells cultured in low glucose DMEM (F3), and cells treated with GLUL siRNA (F4). The results have been calculated and presented as mean ± SD. * P<0.05 is significant.

Recent studies have shown that expression of *GLUL* is maintained in some malignancies, such as luminal breast cancer. The present study approved the results of previous studies in the MCF7 cells, luminal A breast cancer cells, at mRNA and protein levels (Figure 2A-E(Fig. 2)). In this examination, we assessed the expression of the *GLUL* gene in cultures with different amounts of glucose. The data of quantitative RT-PCR revealed the down-regulation of *GLUL* at the low quantity of glucose compared to high and moderate levels of glucose (P-value= 0.0052, 0.0071, respectively) (Figure 2A(Fig. 2)). Similarly, the gene expression study in siGLUL transfected cells demonstrated significant down-regulation of *GLUL* at mRNA and protein levels (p < 0.0001 and 0.0048, respectively) in low glucose cultures (Figure 2B, D, E(Fig. 2)). According to the results, siRNA transfection significantly induced the knockdown of *GLUL* transcripts with ratio = 85 % on MCF7 cells compared to control cells (P-value < 0.0001).

### Impact of levels of glucose and siGLUL on MCF7 cell morphology

Collected data demonstrated that lowered glucose was related to more maturation and spindle-shaped morphology of proliferating cells (**Figure 2F**(Fig. 2)). Also, *GLUL* suppression has been associated with cell shrinkage and death and a significantly decreased number of cells compared to not treated low glucose cultures.

### GLUL suppression, along with decreased levels of glucose, affected the phases of the MCF7 cell cycle

**Figure 3:**
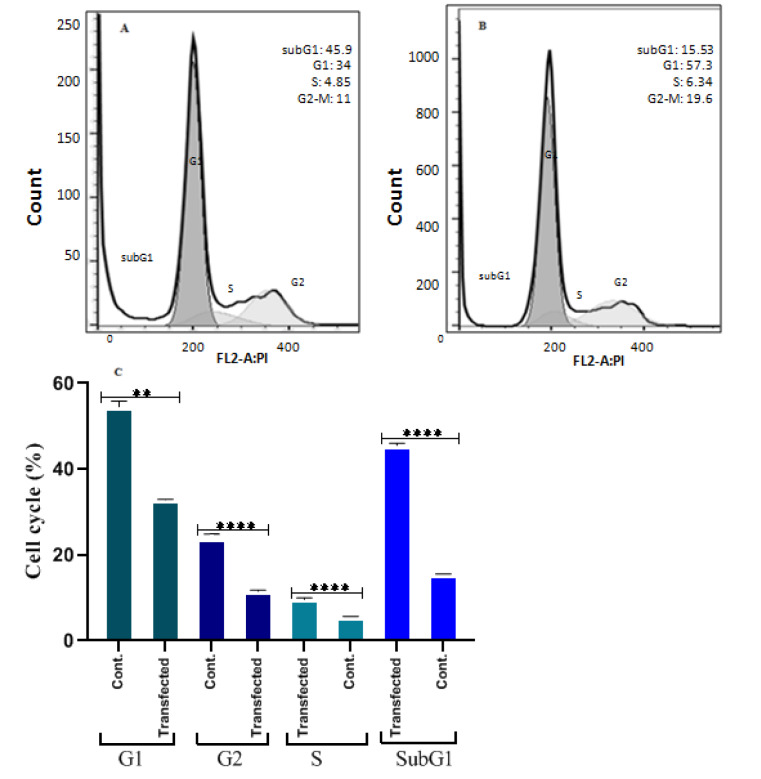
Cell cycle studies A) Not transfected cells B) siRNA transfected cells C) Statistical analysis shows an increased number of cells at the subG1 phase and a decreased number of cells at G2-M phases in transfected cells compared to control cells.

**Figure 4:**
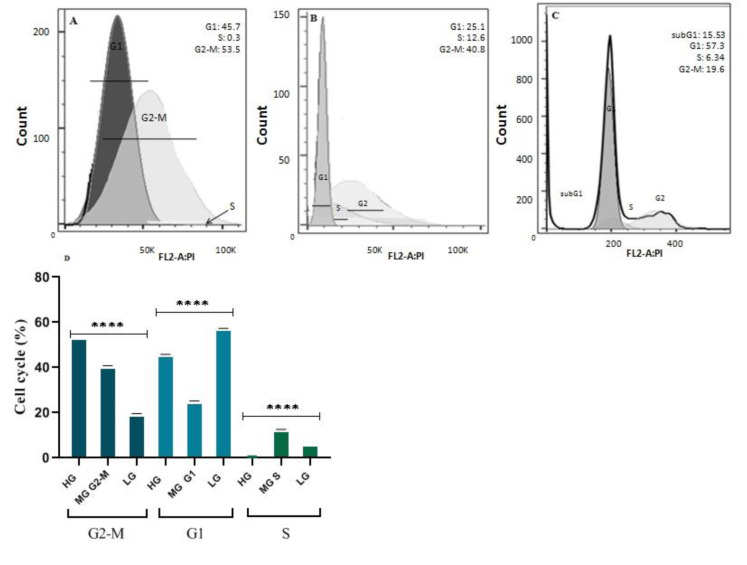
Flow cytometry studies in cells cultured in different amounts of glucose; A) High glucose DMEM, B) Moderate glucose Medium, C) Low glucose DMEM, and D) Statistical analysis of cell cycle phases

The growth inhibitory role of gene knockdown was studied and investigated via flow cytometry. The results demonstrated that GLUL suppression affected the cell population in subG1, G1, S, and G2-M phases (Figure 3(Fig. 3)). In this context, the results showed a significant increase in subG1, which represents late apoptosis, and also a decrease in G1, S, and G2-M in GLUL siRNA transfected cells compared to controls (P<0.0001). As well as flow cytometry studies showed significant cell cycle differences in cultures with various amounts of glucose. In this evaluation, high glucose cultures indicated increased G2-M phases, the cell division stage (p<0.0001) (Figure 4(Fig. 4)).

### GLUL suppression influences migration, and so the invasiveness rate of the MCF7 cells

**Figure 5:**
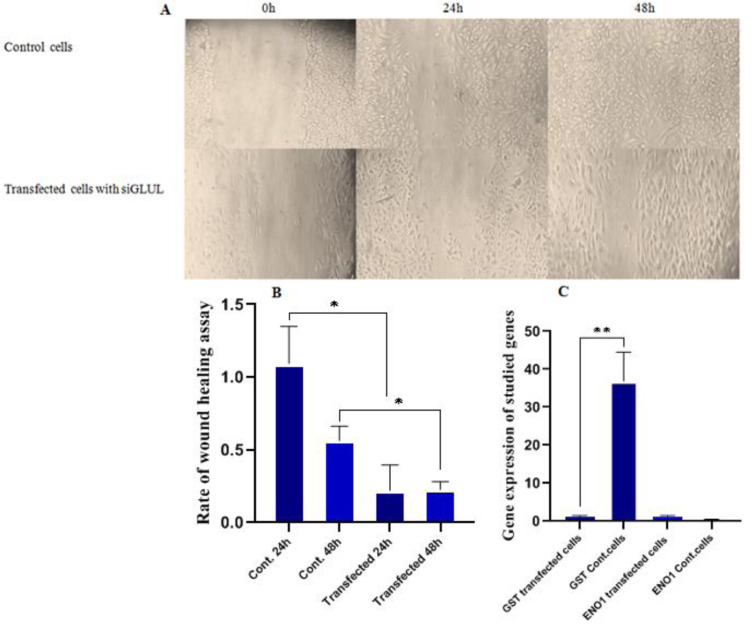
Effects of *GLUL* suppression on migration and invasiveness capacity; A) Wound-healing assay method was run using an inverted microscope (Olympus CKX41) with magnification 10, in control and siGLUL transfected cells, B) Rate of wound healing assay during 0, 24 and 48 hours. Results show a decreased pace of migration in transfected cells compared to control cells, with p=0.0257 and p=0.0227 for 24 h and 48 h, respectively. C) Analysis of *GSTM3* and *ENO1* (p>0.05) expression in control and transfected cells by quantitative RT-PCR 48 h after transfection. The expression of *GSTM3* had decreased in siRNA-transfected cells (p=0.0013).

The effect of GLUL knockdown on migration and aggressiveness capacity of cells was studied utilizing the wound healing assay method. The results revealed the linear relation between suppression of GLUL and decreased rate of migration compared to control MCF7 cells (p=0.0257 and p=0.0227 for 24 h and 48 h, respectively) (Figure 5(Fig. 5)). As well to explore the exact role of GLUL on the invasion power of target cells, the expression of metastatic genes including; Glutathione S transferase Mu3 (GSTM3) and Alfa-enolase (ENO1) were determined using qRT-PCR technique. In this trial, repression of GLUL significantly down-regulated GSTM3 in MCF7 cells (p = 0.0013) but had no significant effect on ENO1 expression (p > 0.05, Figure 5C(Fig. 5)). 

### GLUL knockdown stimulated (programmed cell death) apoptosis

**Figure 6:**
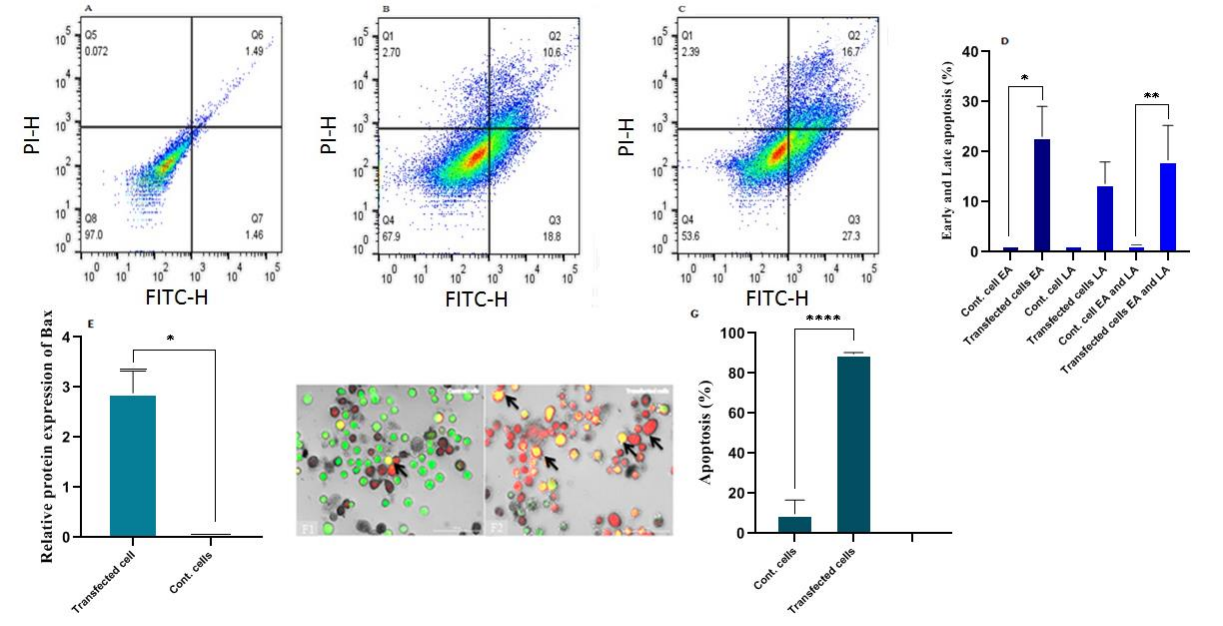
The flow cytometry assessment of apoptosis using FITC-Annexin V and PI staining. Dot plots of apoptosis in A) Control MCF7 cells, B) and C) Transfected MCF7 cells with siGLUL after 48 and 72 hours. In each plot, the viable, early apoptotic, and late apoptotic cells are observable in Q4, Q3, and Q2. D) Statistical analysis shows a significant difference in early and total (early and late) apoptosis with p= 0.0366 and 0.0027). E) Expression of *Bax* in siGLUL transfected and control cells by quantitative RT-PCR 48 h after transfection with increased expression in transfected cells (p=0.0113). F) Morphological studies of apoptosis based on acridine orange/ethidium bromide staining (AO/EtBr) via citation cell imaging system, F1) Control cells, F2) Transfected cells. In this figure, the apoptotic cells are distinguishable by arrows. The nucleus of early apoptotic cells shows yellow and green fluorescence by acridine orange staining, and late apoptotic cells show orange fluorescence by ethidium bromide. G) Based on results, siRNA transfected cells showed many apoptotic cells compared to control cells (p<0.00001). * p < 0.05 was considered significant. EA: Early Apoptosis, LA: Late Apoptosis

To investigate apoptosis, siGLUL-treated and control cells were detached with trypsin and prepared with Annexin V and propidium iodide (PI). As shown in Figure 6A, B, C, and D(Fig. 6), the suppression of *GLUL* resulted in an increased number of early and total (early and late) apoptotic cells in transfected cultures compared to controls (p= 0.0366 and 0.0027, respectively). Also, the cells were evaluated based on morphology differences via acridine orange/ethidium bromide staining. The findings were consistent with the results of Annexin V studies (P < 0.00001). As well as gene expression studies indicated up-regulation of the *Bax* gene in treated cells compared to control cells (P=0.013), while there was no expression of *Bax* in control MCF7 cells (Figure 6E(Fig. 6)).

See also the Supplementary data.

## Discussion

Breast cancer is the most frequent cancer in women all over the world. Metabolic modifications are one of the main hallmarks of cancer cells and can be considered a potential aim in targeted cancer therapy. The present study was planned and conducted to scan the effects of different quantitations of glucose and *GLUL* repression, as the main metabolic pathways, on cell growth in luminal breast cancer, because of the vital role of metabolic reprogramming in cancer cell survival (Cazzaniga and Bonanni, 2015[8]; Faubert et al., 2020[19]; Phan et al., 2014[51]; Schiliro and Firestein, 2021[56]; Sun et al., 2020[62][64]; Sun et al., 2018[63]). Our findings approved the active role of glutamine synthetase in the luminal breast cancer cell line, in concordance with Kung et al.(2011[35]). As well as the effect of various levels of environmental glucose was examined and evaluated on the *GLUL*, *GSTM3*, and *ENO1* gene expression, and based on the results, the *GLUL*, but not *GSTM3*, and *ENO1* are down-regulated by low glucose. Also, flow cytometry assay indicates a decreased G2-M phase of the cell cycle in this situation. We investigated the GLUL siRNA-transfected cells for features including growth, proliferation, and invasion. Briefly, the suppression of *GLUL* has shown a reduced rate of cell growth, proliferation, migration, and increased apoptosis in treated cells. Inducing apoptosis is a critical approach to managing tumors, and we showed knockdown of *GLUL* and limited glucose resources is associated with increased apoptosis, early and late, and up-regulation of the *Bax* gene as a pro-apoptotic factor. We showed that referred situations are associated with an increased cell number in the subG1 phase of flow cytometry assay as an index of programmed cell death. 

Additionally, the knockdown process significantly represses cellular migration and *GSTM3* expression, but it does not affect the *ENO1*. These results are compatible with recent studies where the down-regulation of *GSTM3* results in decreased cell migration and metastasis, increased apoptosis, and improved sensitivity to treatment (Li et al., 2019[38]; Lin et al., 2018[40]). Gene superfamily of glutathione S-transferase (*GST*), including *GSTM3*, as a phase II xenobiotic and drug detoxification enzyme (Deponte, 2013[14]; Di Pietro et al., 2010[16]; Hollman et al., 2016[28]; Sturchio et al., 2008[61]; Tóth et al., 2015[65]; Xie et al., 2015[76]), acts in conjugation with phase I enzymes such as CYPs (Vaish et al., 2020[67]), and this superfamily is also considered critical enzymes in drug resistance, especially in cancers (Singh and Reindl, 2021[58]). In this context, *GSTM3* up-regulation occurs via recruiting and binding SP1, EP 300, and AP-1 transcription factors to its promoter by ER (Estrogen receptor) intermediation in ER-positive breast cancer (Bièche et al., 2004[4]; Lin et al., 2018[40]). Regarding our findings, limited glucose resources and *GLUL* suppression decrease *GSTM3* expression as a known metastatic gene in luminal breast cancer, and these findings further accentuate the significant role of *GSTM3* in invasion potency, a major step in metastasis. Given the detoxification role of this enzyme, reduced *GSTM3* can cause increased cell sensitivity to ROS (reactive oxygen species) such as H_2_O_2_ or chemotherapeutic drugs such as tamoxifen. Regard to recent studies, enhanced aerobic glycolysis and pentose phosphate pathway in malignancies such as breast cancer could decrease ROS production. Considering these results and our findings, a low quantity of glucose may be associated with increased levels of ROS and therefore collaborate in cell death and devastation (Aykin-Burns et al., 2009[2]). Besides, we knocked down *GLUL* and found a lowered expression of *GSTM3* that could improve the context for ROS action. With an overview, we hypothetically imagine *GLUL* suppression with limited glucose, synergistically, could be considered as an impact cancer treating method via ROS enhancement. Also, based on the possible mechanism, this intervention could be used with conventional therapies, including chemotherapy or radiation therapy that also function via ROS, as a new combinatory therapeutic strategy. We also showed up-regulating the *Bax* gene as a result of *GLUL* suppression. In this way, overexpressed *Bax* transfers to mitochondria and stimulates the intrinsic path of apoptosis via releasing cytochrome C and subsequent activation of caspase-3 and caspase-9 (Gao et al., 2001[21]). PI3K/Akt/mTOR signaling pathway is one of the possible paths that can be affected by the glutamine synthetase function (Xiao et al., 2016[75]), and it has also been well-approved to play a critical role in glutamine synthesizing cancerous cells (Paplomata and O'Regan, 2014[49]). As regards recent studies, the PI3K/Akt pathway is known as the crucial path of controlling the expression of pro-apoptotic molecules such as *Bax*, BAD, anti-apoptotic Bcl-2, and other factors including CREB (cAMP response element binding) (CREB) and NF-κB (nuclear factor-kappa B) (Creson et al., 2009[9]; Datta et al., 1997[12]; Du and Montminy, 1998[17]; Kane et al., 1999[33]; Tsuruta et al., 2002[66]). The mTOR acts downstream of Akt and triggers it via mTORC2. Activated Akt presents its role via inhibition of the proteolysis of Cyclin D1/E (Sarbassov et al., 2006[55]; Wander et al., 2011[69]). Based on these explanations and our results, it seems overexpression of the *Bax* has arisen from Akt inhibition following the mTOR repression. According to the role of the glutamine metabolic pathway in luminal breast cancer cells, conversion to glutamate (Glutamine-derived glutamate) and its use in various paths, including protein and glutathione synthesis (Cynober, 2018[10]), transamination reactions (Brosnan, 2000[6]; Cynober, 2018[10]), and other nutrients exchange reactions (Loï and Cynober, 2022[42]; Lu, 2013[44]), stopping glutamine synthesis in luminal breast cancer and other malignancies with a similar metabolism such as glioblastoma multiforme (Obara-Michlewska and Szeliga, 2020[48]) and hepatocellular carcinoma (Long et al., 2011[43]) may be more efficient and considered as a potentially curative strategy. In other words, if these results have been confirmed in the *in vivo* systems (model organisms and clinical trial), providing “a modified diet with limited carbohydrates and suppression of glutamine synthetase, simultaneously, as a “hypothesized multimodal treatment strategy” can be considered a promising therapeutic approach.

Shortly, our findings confirm the critical role of *GLUL* and glycolysis in the metabolic network of luminal breast cancer, alone or together, and also offer principal data on the significance of these two pathways as a potential target therapy against the luminal subtype of breast cancer. 

In conclusion, it seems logical to exploit such multimodal patterns in designing treatment strategies, perhaps as a next-generation treatment strategy, for different types of cancer.

## Declaration

### Acknowledgment

This study has been designed and implemented at the Molecular Genetics Department of the University of Tabriz and Immunology Research Center (IRC), Tabriz, Iran. We thank everyone for assisting us in this project, especially Mr. Hossein Saeidi, laboratory expert of IRC.

### Conflict of interest

The authors declare no potential conflicts of interest. 

### Funding

This study received no grant from funding agencies.

## Supplementary Material

Supplementary data

## Figures and Tables

**Table 1:**
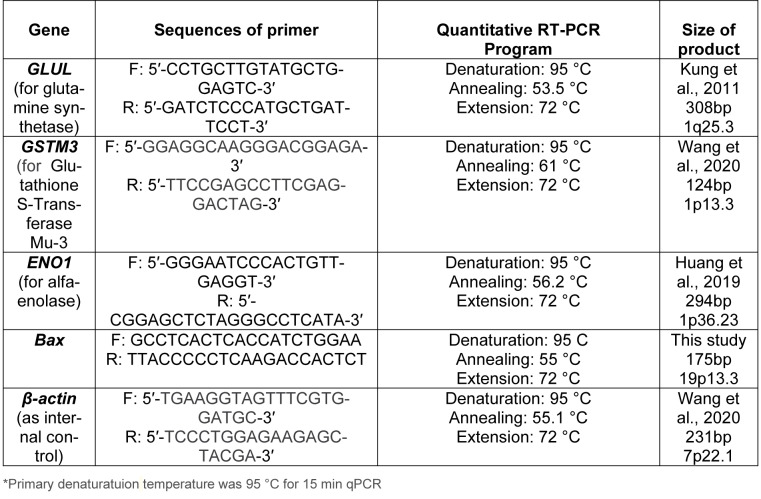
The sequences of the primers and qRT-PCR programs applied in this research

**Figure 1:**
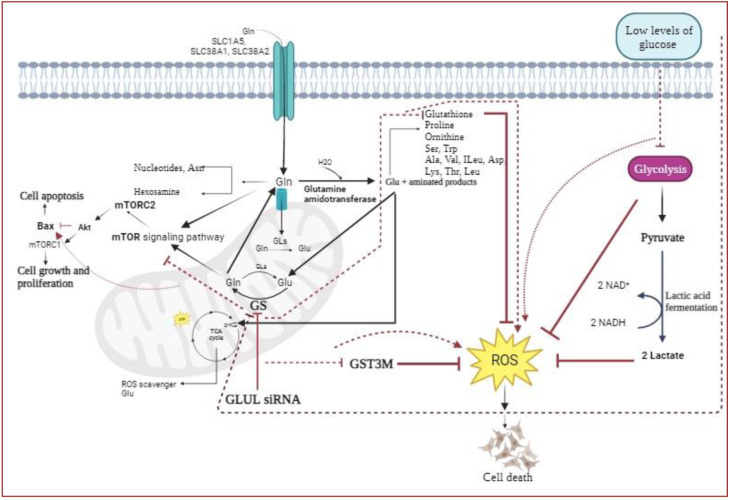
Graphical abstract. This schematic image indicates a possible involved pathway in the current study. Based on our analysis, low quantities of glucose mitigate glycolysis inhibitor and cause increased ROS. Also, GLUL repression down-regulates GST3M, a glutathione transferase. Therefore, ROS molecules accumulated and can harm the cells. As well as the Bax gene, a proapoptotic gene, up-regulates in the result of GLUL knockdown, possibly because of mTOR/ AKT pathway suppression (Т: inhibitory path, ↑: promoting path).
