# Supplementary material for: GLUL gene knockdown and restricted glucose level show synergistic inhibitory effect on the luminal subtype breast cancer MCF7 cells’ proliferation and metastasis
Source: EXCLI J. 2023 Aug 16;22:847–61. doi: 10.17179/excli2023-6287 (PMC10539544; doi:10.17179/excli2023-6287)
Supplement: Supplementary data [file EXCLI-22-847-s-001.pdf]

EXCLI Journal 2023;22:847-861 – ISSN 1611-2156
Received: June 27, 2023, accepted: August 07, 2023, published: August 16, 2023

Supplementary data to:

Original article:

GLUL GENE KNOCKDOWN AND RESTRICTED GLUCOSE LEVEL SHOW SYNERGISTIC INHIBITORY EFFECT ON THE LUMINAL SUBTYPE BREAST CANCER MCF7 CELLS' PROLIFERATION AND METASTASIS

Arezu Karimpur Zahmatkesh[1], Mohammad Khalaj-Kondori[1*], Mohammad Ali Hosseinpour Feizi[1], Behzad Baradaran[2]

1 Department of Animal Biology, Faculty of Natural Sciences, University of Tabriz, Tabriz, Iran
2 Immunology Research Center, Tabriz University of Medical Sciences, Tabriz

* **Corresponding author:** Mohammad Khalaj-Kondori, Department of Animal Biology, Faculty of Natural Sciences, University of Tabriz, Tabriz, Iran. Tel. +984133392674; E-mail: khalaj@tabrizu.ac.ir

https://dx.doi.org/10.17179/excli2023-6287

This is an Open Access article distributed under the terms of the Creative Commons Attribution License (http://creativecommons.org/licenses/by/4.0/).

EXCLI Journal 2023;22:847-861 – ISSN 1611-2156
Received: June 27, 2023, accepted: August 07, 2023, published: August 16, 2023

Figure 2A, B, C, D: Expressions of GLUL (glutamine synthetase) mRNA in breast cancer MCF7 cells in a growth medium with different levels of glucose and siGLUL (siRNA against GLUL) transfected cultures and also western blotting trials of glutamine synthetase have been indicated.

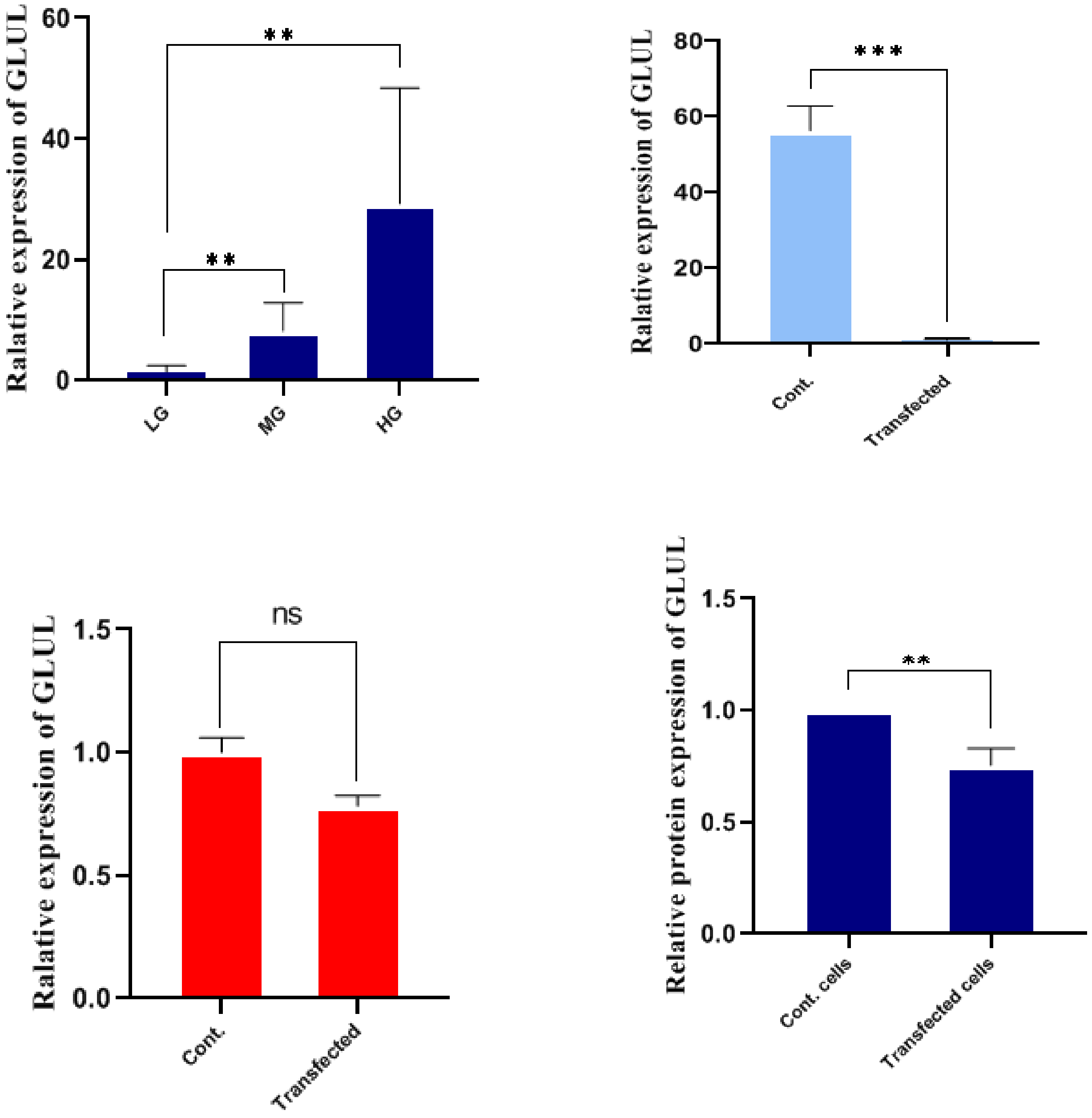

EXCLI Journal 2023;22:847-861 – ISSN 1611-2156
Received: June 27, 2023, accepted: August 07, 2023, published: August 16, 2023

Raw data to Figure 2A, B, C, D: The raw data indicating fold changes of studied genes of individual qRT-PCR include GLUL (Glutamate Ammonia Ligase), GSTM3 (glutathione-S-transferase Mu3), ENO1 (alfa-enolase), and Bax genes.

Genes	Experiments	Transcript Fold change ($2^{-\Delta\Delta CT}$) in control cells	Transcript Fold change ($2^{-\Delta\Delta CT}$) in transfected cells
**GLUL HG**	Experiment 1	23.52299738	
	Experiment 2	38.00190931	
	Experiment 3	59.63138748	
	Experiment 4	34.96888765	
	Experiment 5	7.878931543	
	Experiment 6	11.53543097	
	Experiment 2	12.44936282	
	Experiment 3	4.556733509	
	Experiment 4	12.02529182	
	Experiment 2	0.214344009	
	Experiment 3	0.971307496	
	Experiment 4	3.267075964	
	Experiment 5	1.988940337	
	Experiment 6	0.839731493	
**GLUL LG**	Experiment 1	63.55791971	1.375542
	Experiment 2	50.91433496	0.726986
	Experiment 3	54.56863307	1.051264
**GSTM3**	Experiment 1	44.32350298	0.742261785
	Experiment 2	29.04061297	1.347233577
	Experiment 3	37.27147477	0.53338786
**ENO1**	Experiment 1	0.742261785	0.334481889
	Experiment 2	1.347233577	0.158219574
	Experiment 3	0.53338786	0.539614118
**Bax**	Experiment 1	3.193194	1
	Experiment 2	2.575763	1
	Experiment 3	1.29899997	1

EXCLI Journal 2023;22:847-861 – ISSN 1611-2156
Received: June 27, 2023, accepted: August 07, 2023, published: August 16, 2023

Raw data to Figure 2A, B, C, D: The raw data indicating fold changes **mean** of studied genes of individual experiments include GLUL (Glutamate Ammonia Ligase), GSTM3 (glutathione-S-transferase Mu3), ENO1 (alfa-enolase), and Bax genes.

	Genes for cell cultures in different levels of glucose	Transcript Fold change ($2^{-\Delta\Delta CT}$)
**1**	GLUL in high glucose DMEM	29.25659
**2**	GLUL in moderate glucose DMEM	8.249564
**3**	GLUL in low glucose DMEM	1.417058
**-**	-	-
**4**	GLUL in high glucose DMEM control cells	1.00086494
**5**	GLUL in high glucose DMEM transfected cells	0.782437885
**6**	GLUL in low glucose DMEM control cells compared to transfected cells	56.34696258
**7**	GLUL in low glucose DMEM transfected cells	1.051264

	Genes for siRNA transfected and not transfected cells	Transcript Fold change
**1**	GLUL in siGLUL transfected cells	1.051264
**2**	GLUL in control cells	56.34696258
**3**	GSTM3 in siGLUL transfected cells	1.056901246
**4**	GSTM3 in control cells	36.87853024
**5**	ENO1 siGLUL transfected cells	1.044747681
**6**	ENO1 in control cells	0.344105194
**7**	Bax in siGLUL transfected cells	2.8844785
**8**	Bax in control cells	1

	Protein for siRNA transfected cells in low glucose cultures	Protein Fold change
	GS (glutamine synthetase) transfected cells	0.678212216
	GS (glutamine synthetase) control cells	1

EXCLI Journal 2023;22:847-861 – ISSN 1611-2156
Received: June 27, 2023, accepted: August 07, 2023, published: August 16, 2023

Raw data to Figure 2E and F: The captured image from cell morphology and the western blotting trial has been indicated.

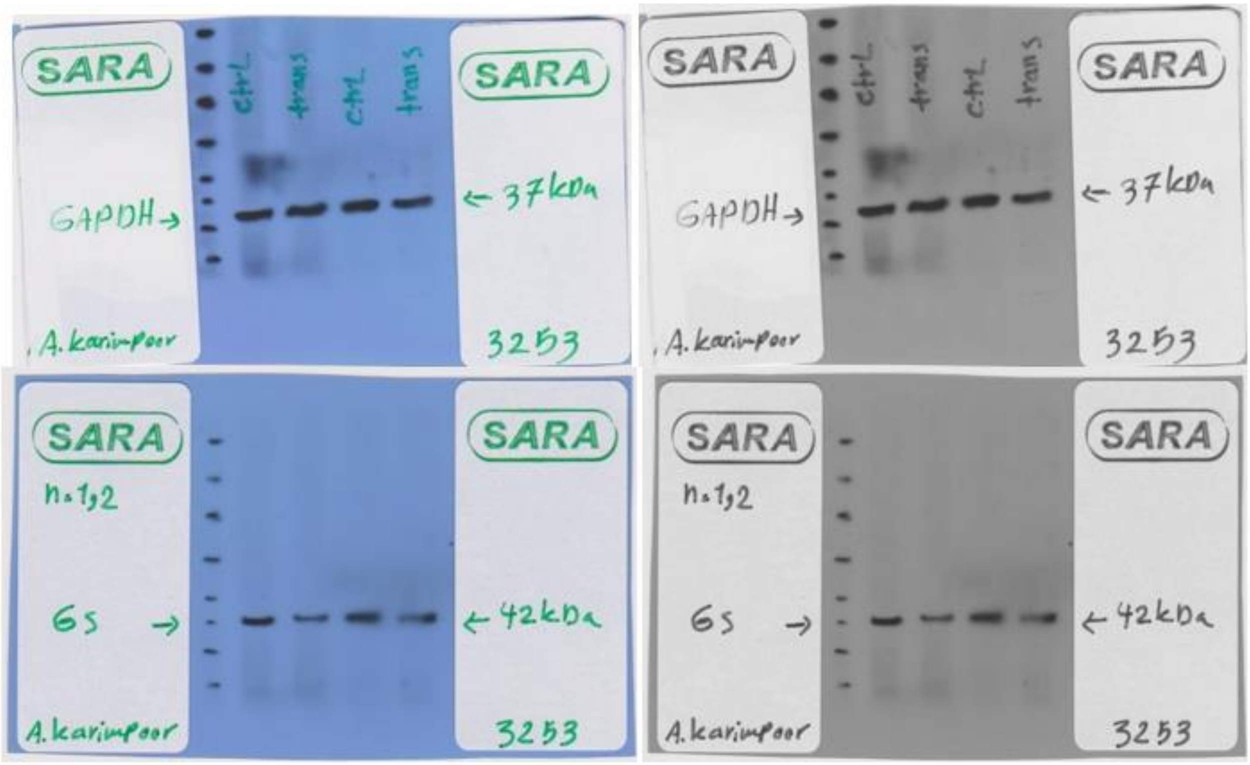

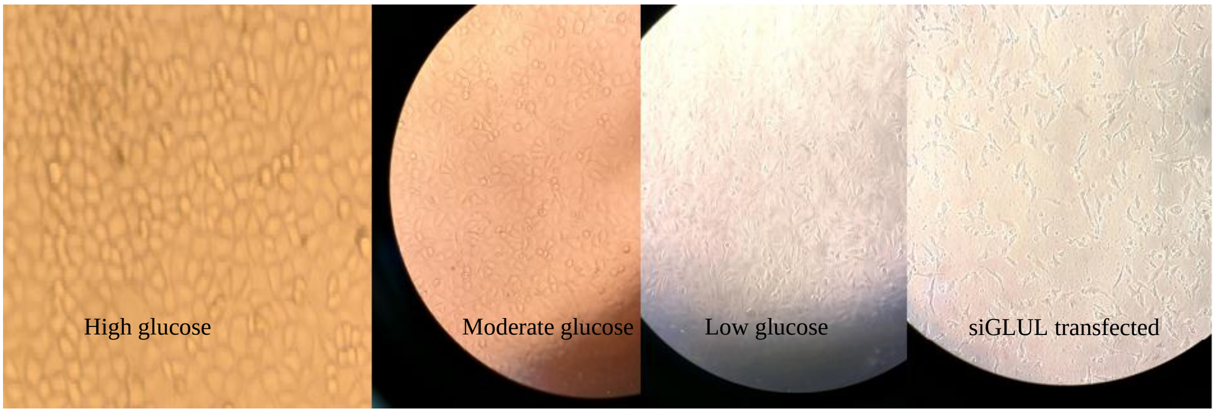

EXCLI Journal 2023;22:847-861 – ISSN 1611-2156
Received: June 27, 2023, accepted: August 07, 2023, published: August 16, 2023

Figures 3 and 4: Cell cycle studies via flow cytometry assay

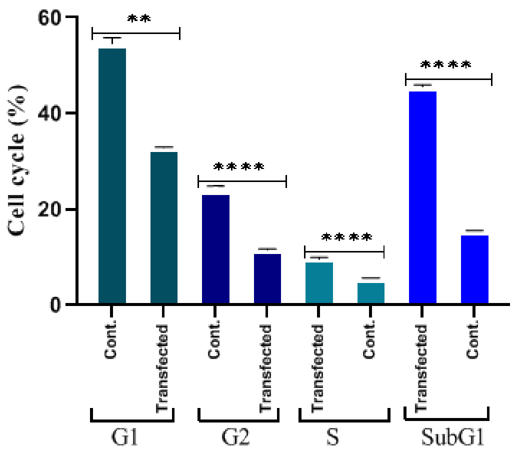

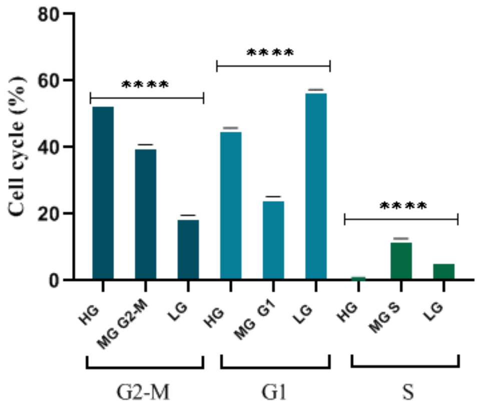

EXCLI Journal 2023;22:847-861 – ISSN 1611-2156
Received: June 27, 2023, accepted: August 07, 2023, published: August 16, 2023

Raw data to Figures 3 and 4: The flow cytometry diagrams for cells in different conditions, including various levels of glucose and siRNA transfected cultures, have been shown in this section. The data have been used to statistical analysis, and the results are shown in the following table.
HG: high glucose, LG: low glucose, MG: moderate glucose

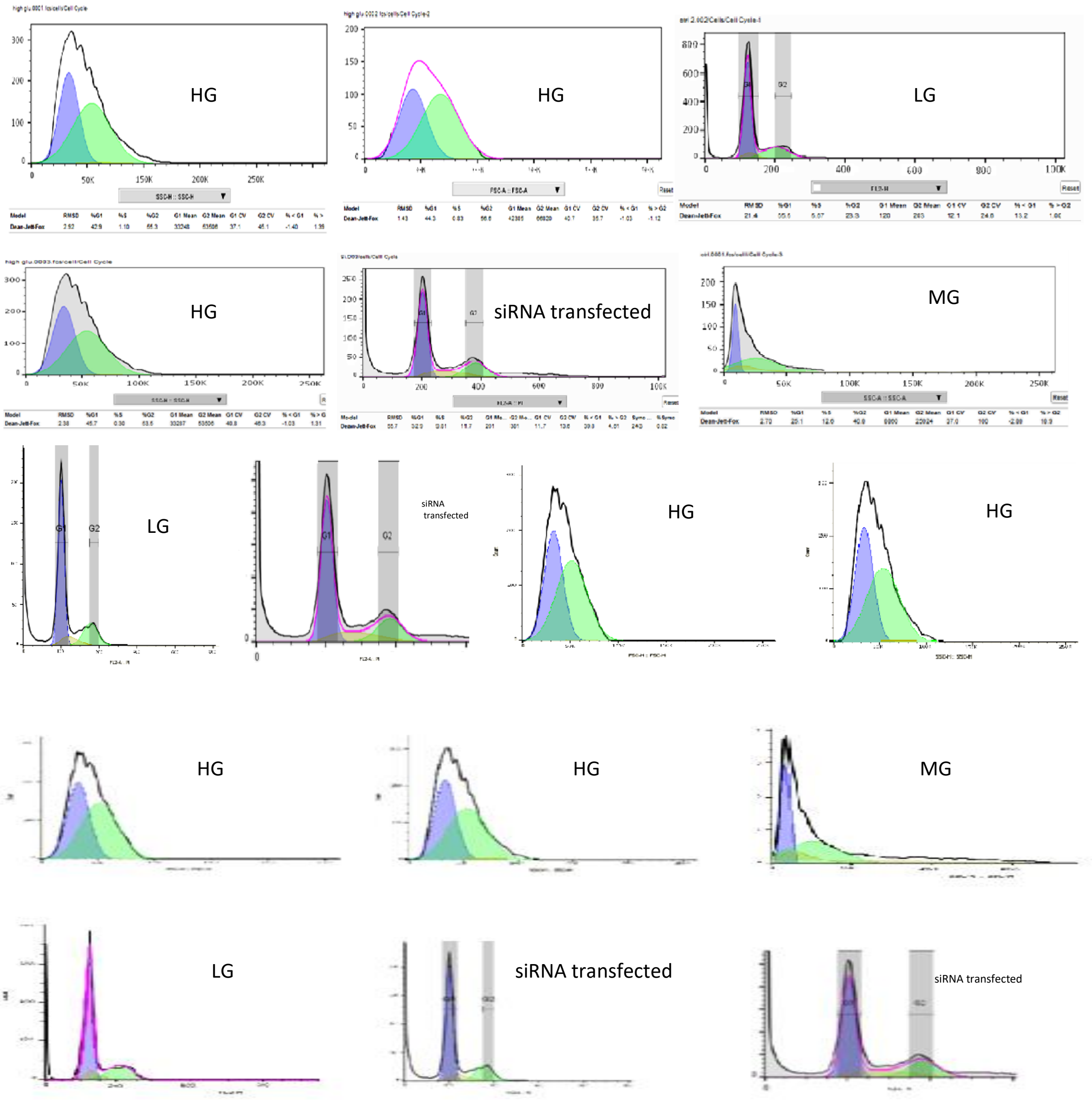

EXCLI Journal 2023;22:847-861 – ISSN 1611-2156
Received: June 27, 2023, accepted: August 07, 2023, published: August 16, 2023

The raw data of Figure 3 and 4 are showing the average of cell numbers in different phases of cell cycle and also the result of statistical analysis.

**Cell cycle phases**	**subG1**	**G1**	**S**	**G2-M**
**High glucose Cell number mean %**	-	45.75 %	0.4 %	53.5 %
**Moderate glucose Cell number mean %**	-	25.15 %	12.55 %	40.75 %
**Low glucose Cell number mean %**	15.53 %	57.3 %	6.355 %	19. 55 %
**siGLUL transfected cells Cell number mean %**	45.8 %	32.85 %	9.82 %	11.65 %
**Not transfected Cell number mean %**	15.525 %	54.25 %	5.68%	23.25 %
**High glucose vs moderate glucose P-value**	**-**	**<0.0001**	**<0.0001**	**<0.0001**
**High glucose vs low glucose P-value**	**<0.0001**	**<0.0001**	**<0.0001**	**<0.0001**
**Low glucose vs moderate glucose P-value**	**-**	**<0.0001**	**<0.0001**	**<0.0001**
**siGLUL transfected vs not transfected (control) cells P-value**	**-**	**=0.0034**	**<0.0001**	**<0.0001**

EXCLI Journal 2023;22:847-861 – ISSN 1611-2156
Received: June 27, 2023, accepted: August 07, 2023, published: August 16, 2023

Raw data to Figure 5A: Examples of captured images of scratches in hours 0, 24, and 48 in siRNA transfected and control cultures have been demonstrated. Collected data were analyzed using Fiji-ImageJ, and healing rate was determined with the mentioned formula.

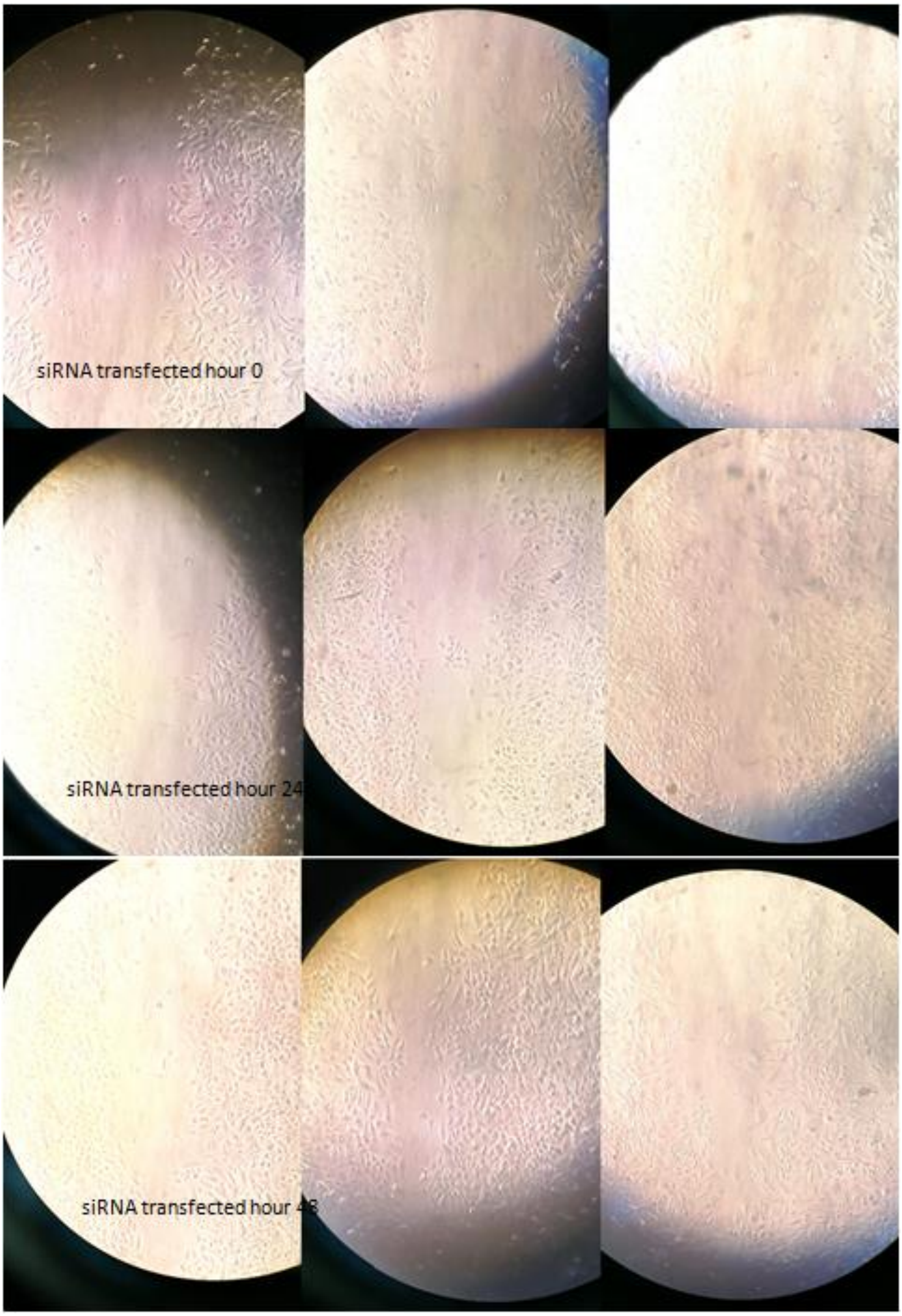

EXCLI Journal 2023;22:847-861 – ISSN 1611-2156
Received: June 27, 2023, accepted: August 07, 2023, published: August 16, 2023

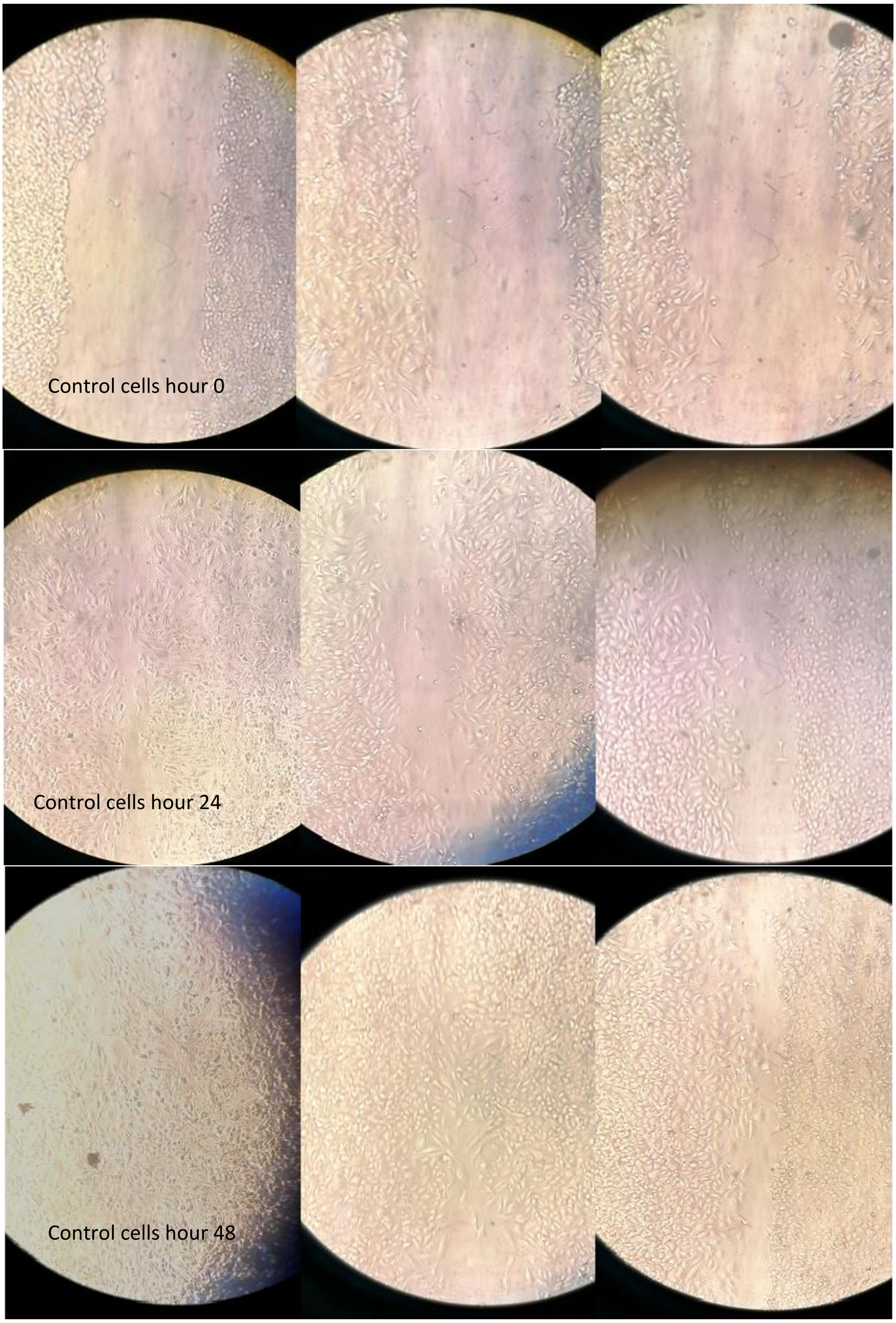

EXCLI Journal 2023;22:847-861 – ISSN 1611-2156
Received: June 27, 2023, accepted: August 07, 2023, published: August 16, 2023

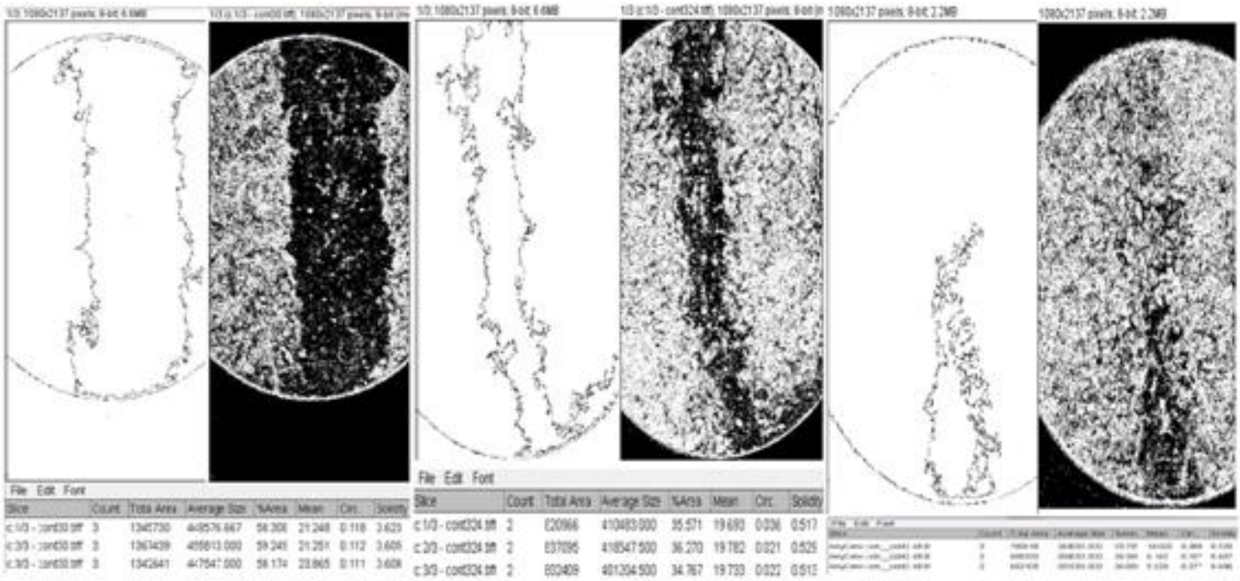

Fiji-ImageJ software was used to anlyze the collected data of wound healing assay**.**
The formula was used to calculate the rate of healing.

$R_{M} = W_i – W_f /t$

W_i = initial wound width (nm)
W_f = final wound width (nm)
t = duration of migration (hour)

EXCLI Journal 2023;22:847-861 – ISSN 1611-2156
Received: June 27, 2023, accepted: August 07, 2023, published: August 16, 2023

Figure 5B and C: Effects of *GLUL* suppression on migration and invasiveness capacity

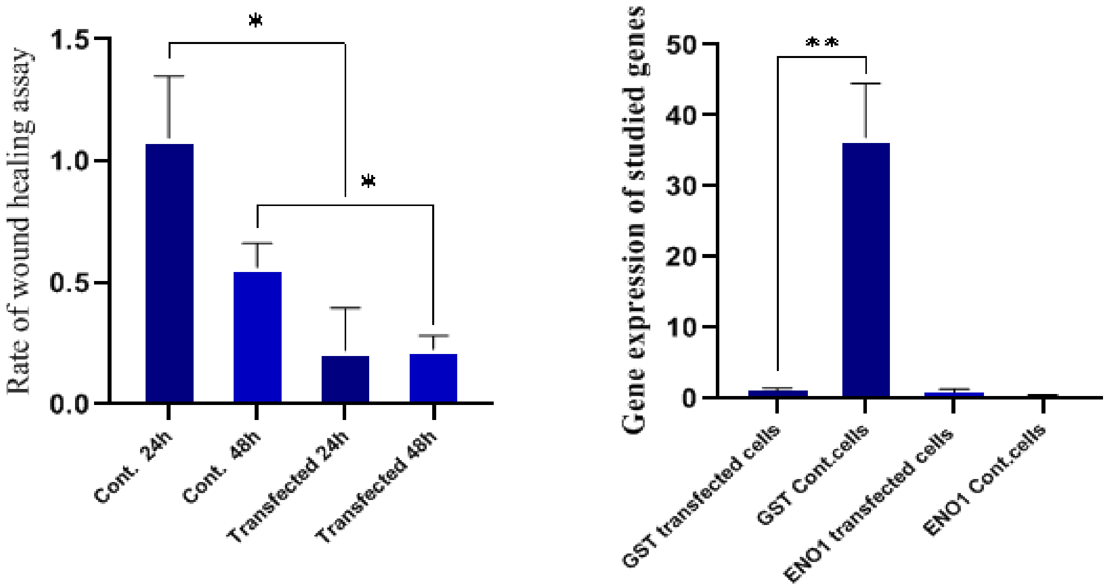

Raw data to Figure 5B: The average rate of wound healing assay was determined after 24 and 48 hours

The average rate of wound healing assay (migration rate) (nm/hour)	After 24 hours	After 48 hours
**siGLUL transfected cells**	0.2215nm/h	0.227nm/h
**Not transfected Cell**	1.0943333nm/h	0.564666nm/h
**siGLUL transfected vs not transfected (control) cells P-value**	**0.0257**	**0.0227**

Raw data to Figure 5C: The raw data indicating fold changes of studied metastatic genes including GSTM3 and ENO1

	Metastatic genes	Transcript Fold change
1	GSTM3 in siGLUL transfected cells	1.056901246
4	GSTM3 in control cells	36.87853024
5	ENO1 siGLUL transfected cells	1.044747681
6	ENO1 in control cells	0.344105194
7	GSTM3 in transfected cells vs control cells P-value	**0.0013**
8	ENO1 in transfected cells vs control cells P-value	0.1188

EXCLI Journal 2023;22:847-861 – ISSN 1611-2156
Received: June 27, 2023, accepted: August 07, 2023, published: August 16, 2023

Raw data to Figure 6A, B and C: Examples of flow cytometry diagrams for cell apoptosis in siRNA transfected and control cultures have been indicated in this section. The data have been used for statistical analysis, and the results are shown in the following tables, including apoptosis assay based on Annexin V and acridine orange/ethidium bromide, respectively.

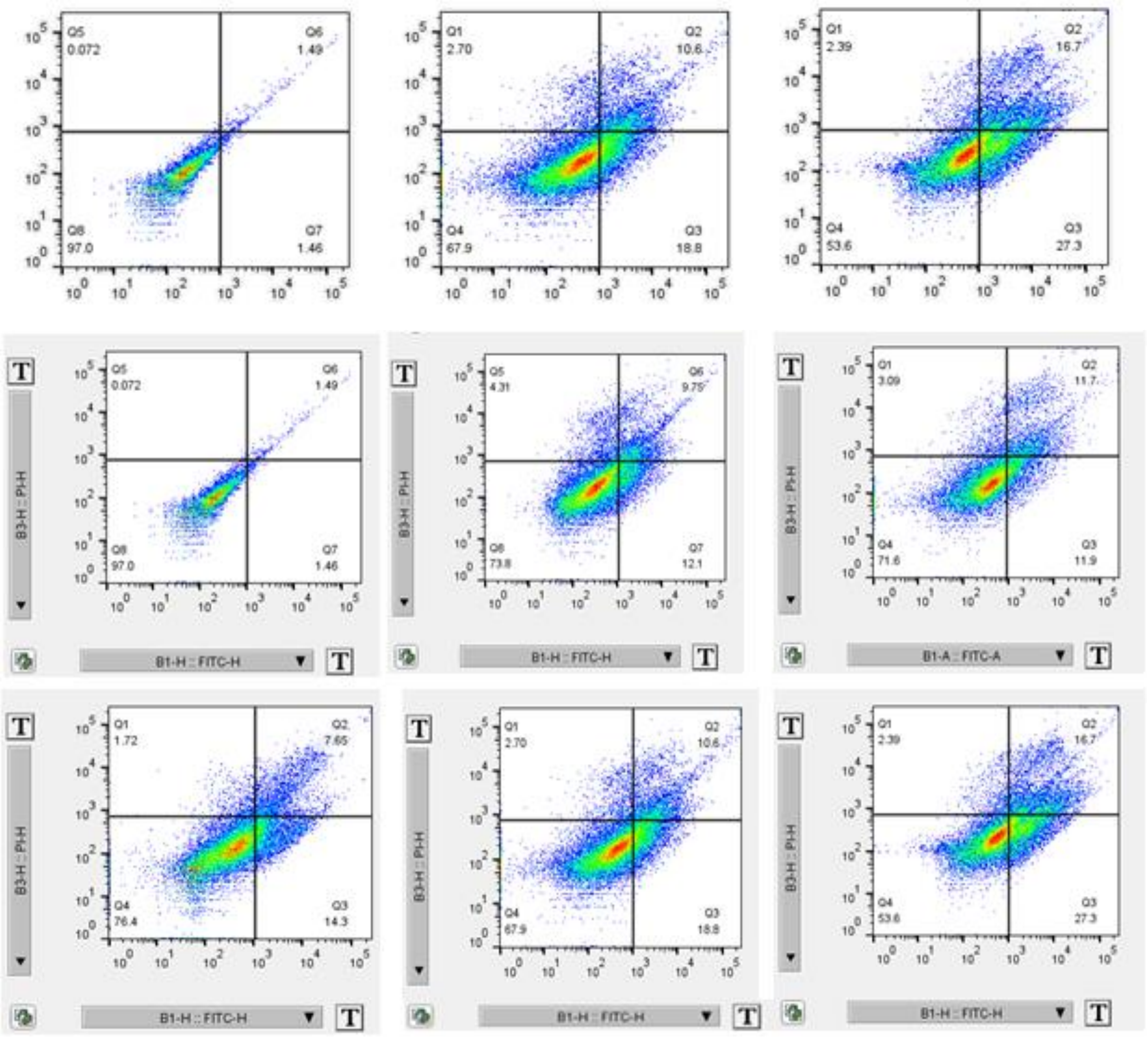

EXCLI Journal 2023;22:847-861 – ISSN 1611-2156
Received: June 27, 2023, accepted: August 07, 2023, published: August 16, 2023

Figure 6D: The flow cytometry assessment of apoptosis using FITC-Annexin V and PI staining

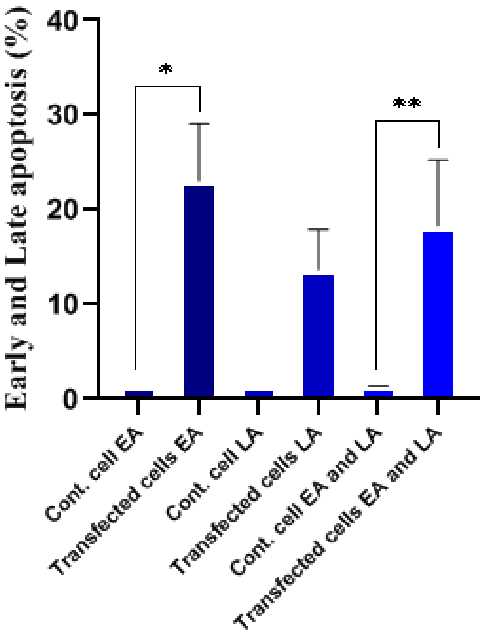

Raw data to Figure 6D: Raw data of apoptosis based on Annexin V / PI

**Cells**	**Early apoptotic cells (%)**	**Late apoptotic cells (%)**	**Early and late apoptosis, to-tally**
**siGLUL transfected cells**	23.05	13.65	18.35
**Not transfected Cell**	1.46	1.48	1.47
**siGLUL transfected vs not transfected cells P-value**	**0.0366**	0.0575	**0.0027**

Raw data to Figure 6E: Raw data demonstrating the fold changes of the studied pro-apoptotic gene (Bax)

	**Pro apoptotic gene**	**Transcript Fold change**
1	Bax in siGLUL transfected cells	2.8844785
2	Bax in control cells	1

Raw data to Figure 6G: Raw data of apoptosis studies based on acridine orange/ethidium bromide

	**Cells**	**Apoptotic cells (%)**
1	Transfected cultures	89.53
2	Not transfected cultures	9.80133

Moderate glucose DMEM preparation:
Production of DMEM culture medium with medium glucose (2 g) is done directly by adding 1 g (5.5 mM) of glucose powder (DNAbiotec, South Africa) with 180.156 g/mol molecular weight to 1 liter of low-glucose DMEM.
